# Accelerated recovery using magnesium ibogaine: characterizing the subjective experience of its rapid healing from neuropsychiatric disorders

**DOI:** 10.1038/s44184-026-00185-7

**Published:** 2026-01-31

**Authors:** Clayton Olash, Derrick Matthew Buchanan, Randi Brown, Afik Faerman, Kirsten Cherian, George Lin, David Spiegel, James J. Gross, Nolan Williams

**Affiliations:** 1 Stanford Brain Stimulation Lab. Department of Psychiatry and Behavioral Sciences, Stanford University School of Medicine, Palo Alto, CA USA; 2 Medical University of South Carolina, Department of Psychiatry, Charleston, SC USA; 3 Department of Psychiatry and Behavioral Sciences, Stanford University School of Medicine, Palo Alto, CA USA; 4 Stanford Psychophysiology Laboratory. Department of Psychology, Stanford University, Palo Alto, CA USA

**Keywords:** Human behaviour, Psychology, Neuroscience, Cognitive neuroscience, Synaptic plasticity, Brain injuries

## Abstract

Magnesium-ibogaine, a formulation combining ibogaine with pre- and post-treatment magnesium, was recently found to yield rapid clinical improvements in U.S. Special Operations veterans with TBI and PTSD. Yet, its therapeutic phenomenology during such healing is unknown. We analyzed post-session narratives from 30 male veterans who, after a single open-label magnesium-ibogaine treatment, answered three open-ended questions. A constructivist grounded-theory approach identified four recurrent experiential domains: dialogic trauma re-appraisal marked by guided replay of autobiographical memories; altered-self and mystical connectedness; emotional resolution with surges of forgiveness, love, and renewed purpose; and embodied healing, a vivid sense of neural repair accompanied by cognitive clarity and somatic relief. Together, these themes portray an accelerated, self-directed psychotherapeutic process that dovetails with previously reported improvements in this same cohort, suggesting mind–body mechanisms involving rapid neuroplastic change and highlighting its potential to inform novel approaches to trauma and TBI.

## Introduction

Ibogaine, a naturally occurring indole alkaloid used ritually in West‑Central Africa^1^, has recently re‑emerged as a candidate therapy for neuropsychiatric disorders^2,3^. At least 7 observational trials of ibogaine have identified rapid reductions in cravings and/or withdrawal symptoms for opioid misuse^4,5^, and, more recently, drastic improvements in post‑traumatic stress disorder (PTSD), depression, and self-reported disability in the context of traumatic brain injury (TBI)^2,3^. Despite these clinical signals, its lived phenomenology and its link to therapeutic change remain unclear.

Contemporary psychedelic research suggests that the quality of the subjective experience often predicts clinical outcome^6–8^. Yet ibogaine is pharmacologically distinct from classic 5‑HT2A agonists^9^ and is reputed to induce an “oneiric,” or dream‑like state^10,11^ that may engage different psychological mechanisms. To date, most ibogaine research has been quantitative, and the limited qualitative work available has centered on broad experiential themes in substance‑use disorders^12,13^. Although systematic qualitative work on ibogaine remains limited, several prior studies have examined its subjective and therapeutic phenomenology. Rodríguez-Cano and colleagues^14^ conducted a qualitative analysis of underground ibogaine use for substance-use disorders, identifying themes of catharsis, autobiographical review, and renewed purpose. Similarly, Davis et al.^15^ used mixed methods to explore persisting positive effects following ibogaine detoxification, reporting increased self-understanding and emotional release. Other accounts have likewise emphasized symbolic, visionary, and psychospiritual dimensions of the experience^11,16,17^.

The present study extends this literature by applying a constructivist grounded-theory approach to a distinct clinical population—U.S. Special Operations veterans with trauma and traumatic brain injury—whose psychological recovery and neurocognitive performance has been objectively documented in Cherian et al. (2024)^2^. By pairing a constructivist grounded‑theory approach^18,19^ with a cohort already established to exhibit unusually rapid, multi‑domain healing, we aim to illuminate the subjective processes that may underlie these objective outcomes and to extend qualitative psychedelic research into a uniquely treatment‑resistant clinical context.

## Methods

This study was approved by the Stanford University Institutional Review Board and pre-registered at clinicaltrials.gov (NCT04313712). This study addresses Aim 5.4 from the Open Science Framework: “To conduct a qualitative analysis of narrative mystical experiences in this observational study of individuals with a history of TBI”.

The current study involved analysis of data collected as part of a prospective, open-label investigation of the safety and clinical efficacy of magnesium–ibogaine therapy in veterans with traumatic brain injuries (Cherian et al., 2024). After having independently scheduled themselves for ibogaine treatment at a clinic in Mexico, potential participants were introduced to the study and referred if interested. Each participant was screened for the study according to the inclusion and exclusion criteria outlined in Cherian et al. (2024). Written informed consent was obtained from all participants.

### Participant demographic data

Participants were 30 male Special Operations Veterans with a history of TBI. The participant age range was 36–58 (mean age 44.9 ± 7.5). Participants had experienced 5.5 ± 3 combat deployments and an average of 38.6 ± 52.4 mainly mild TBI, most often related to blasts. Of the 30 participants, at baseline, 23 met the criteria for Posttraumatic Stress Disorder (PTSD), 15 for Major Depressive Disorder (MDD), 14 for an anxiety disorder, 15 for Alcohol Use Disorder (AUD), and 6 for another substance use disorder (SUD). Please see Cherian et al. (2024) for additional information on the demographics of study participants.

### Study protocol

Participants visited Stanford University for neuropsychological, neuroimaging, and other medical evaluations at baseline (two to three days before treatment), immediate post (three to four days following treatment), and one-month post-treatment. Participants traveled to the Ambio Life Treatment Center in Tijuana, Mexico, for the administration of magnesium-ibogaine in the context of a healing retreat.

**Fig. 1:**
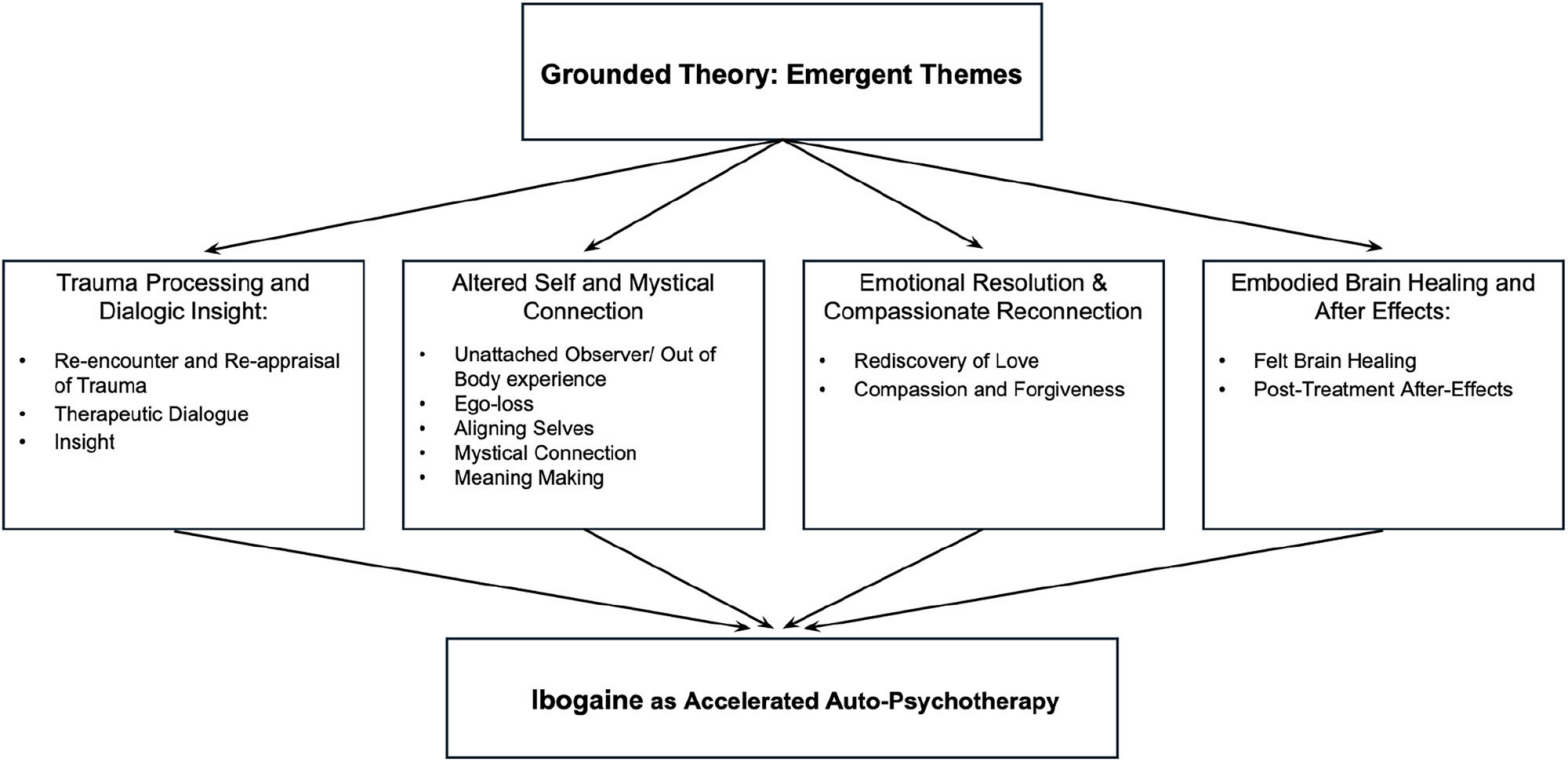
Summary of themes that emerged from grounded theory analysis as they relate to accelerated auto-psychotherapy.

The treatment protocol encompassed 5 days at the Ambio Life Sciences clinic and involved a group format with a maximum of 5 participants. Day 1 included arrival, medical assessment, and preparatory ceremonial activities. Day 2 involved an 8-hour period of fasting, an intravenous infusion of 1 g magnesium sulfate, and an oral gastrointestinal protective agent (1–2 h preceding ibogaine), all undertaken in preparation for the ibogaine dosing. The ibogaine used in this protocol was synthesized in South Africa by Cape Analytical Service Laboratories (ibogaine hydrochloride, 98% pure, not a GMP product) (Fig. 1).

Ibogaine dosing began in the evening of Day 2 with a test dose of 2–3 mg/kg. After ~40 min, additional doses were administered across a 2-h period, with the total dose administered not exceeding 14 mg/kg. One participant received an additional dose of 4 mg/kg 12 h following the primary dose; this occurred at the discretion of clinic personnel due to insufficient treatment intensity. Following dosing, participants underwent continued monitoring for 72 h as ibogaine’s effects can persist throughout this timeframe. Twelve hours after the final ibogaine dose, an intravenous dose of magnesium sulfate, oral and intravenous antioxidants, and metabolic supporting agents were administered.

Ibogaine administration took place in a large room with mats spread on the floor. Although administered in a common space, the ibogaine experience was largely individual as participants spent the majority of the treatment session lying prone on a mat with eyeshades. Medical staff were present at a 1:2 staff-to-patient ratio for monitoring and management. No specific coaching or psychological support was provided during the ibogaine experience period. On Day 4, after ibogaine’s acute effects had largely subsided, participants undertook integration activities, which included group or individual sessions with coaches to process the psychedelic experience as well as opportunities to engage in the following wellness activities: sweat lodge, massage, yoga, reiki, breath work, and meditation. Participants returned to Stanford University for follow-up assessment on Day 5.

Additional therapeutic support during study participation was provided by VETS, Inc., pairing Veterans with licensed therapists who had experience coaching patients undergoing psychedelic therapy. Interaction with these therapists encompassed virtual pretreatment preparatory sessions (coaching on intention-setting, treatment expectations, and managing anxiety) and posttreatment integration sessions (processing emotions and integrating insights). Please refer to Cherian et al. (2024) for full details of methods and clinical outcomes.

### Data collection

At the immediate post-treatment time point, participants were asked to respond to open-ended questions (see Supplement 1), and answers were recorded in REDCap electronic data capture tools hosted at Stanford University^20,21^. REDCap (Research Electronic Data Capture) is a secure, web-based software platform designed to support data capture for research studies, providing (1) an intuitive interface for validated data capture; (2) audit trails for tracking data manipulation and export procedures; (3) automated export procedures for seamless data downloads to common statistical packages; and (4) procedures for data integration and interoperability with external sources. Each participant wrote or dictated responses to these questions. All questions were completed within an average of 13.3 days (range 1-86 days) after their ibogaine experience.

All participants who submitted written narrative reflections in response to 3 questions (see Supplement 1) post-treatment were included in the analysis. We used a purposeful sampling strategy in that the analytic focus was guided by our research aim: to characterize subjective processes related to psychological healing and therapeutic transformation.

Data were analyzed using a constructivist grounded theory approach. A constructivist grounded‑theory position^19^ posits that meaning is co‑constructed between participants’ texts and researchers’ interpretive lenses. Coding was carried out in parallel by two researchers (CO & DB), followed by consensus meetings to resolve discrepancies and triangulate interpretations. Member checking was not feasible due to the anonymous, time-limited post-treatment survey design; participants were not recontacted. At the time of data collection, CO was a resident physician in psychiatry at the Medical University of South Carolina, and DB was a postdoctoral scholar in Psychiatry at Stanford Medical School. Both coders had prior clinical exposure to Veterans but no personal ibogaine experience. The coders also had no interaction with the participants. These researchers independently conducted open coding, generating preliminary codes from the raw narratives. These codes were then collaboratively refined and grouped into broader thematic categories through an iterative and comparative process involving the other co-authors on this paper. Although we did not formally define or measure “saturation,” analysis continued until thematic coherence was achieved around our primary focus: the psychological and psychotherapeutic processes reported by participants. We did not aim to capture all possible themes within the dataset but prioritized those most relevant to the study’s conceptual focus on healing, transformation, and therapeutic insight.

Data was extracted from REDCap and transferred into a HIPAA-compliant cloud server for further analysis. Verified de-identified data was transferred into Delve, a software package specialized for qualitative thematic analyses (*Qualitative Data Analysis Learning Center — Delve*, n.d^22^.).

## Results

Below, we present the final emergent themes from our grounded theory analysis. Each of the primary themes is summarized based on participants’ descriptions and accompanied by sub-themes and examples of relevant direct quotations from participants. Any possible identifying information was redacted to preserve anonymity. These themes represent different aspects of the participants’ subjective experience of ibogaine. For brevity, one or two quotes per theme have been included; please refer to Supplement 2 for additional supporting quotations per theme.

### Trauma processing and dialogic insight

A major theme involved *re-encountering and re-appraising traumatic memories*. Traumatic, sometimes previously repressed, experiences replayed with radically altered affect—often suffused with love, forgiveness, and acceptance—leading to catharsis. As one participant described:

“I remembered the door gunner from the helicopter shoot-down. I carried his body out. The stains were on my uniform; it haunted me for all these years… When I remembered to look for him, he appeared. He was smiling and happy. It released me, I felt that pain go away, and it was immense”.

Another participant provided an especially clear instance of resurfacing long-buried trauma:

“During my journey on Ibogaine, I was able to uncover something that I had buried so deep that decades of therapy with the best therapists in the world would have never uncovered…. My mind blacked it all out except for a couple of fleeting memories in order to survive. I had carried this burden around with me for over 30 years, not knowing what was wrong with me, and it was the cause of my drinking to excess and indulging in food in order to cope. After Ibogaine, all the past trauma associated with that event is gone. The weight of the world on my shoulders is gone… Ibogaine saved my life!!!”

Participants also frequently described a form of *therapeutic dialog*, in which ibogaine was experienced as an interactive guide or teacher providing lessons, moral review, and direction. One wrote:

“The voice that spoke to me in the darkness spoke out again, asking me if I understood the lessons I was being provided”.

Relatedly, many engaged in *self-reflective insight* regarding their own maladaptive patterns or relational dynamics. As one participant explained:

“I asked how I could be a better husband to my wife. I had a long, deep conversation with my higher consciousness on this. We went around and around, and eventually, I realized that I needed to love her without guilt”.

### Altered self and mystical connection

Participants commonly reported experiences involving *detached, observer-like awareness*, describing perceptions of witnessing events unfold from an objective vantage point:

“It felt more like my ego was suspended, and I was completely a witness and not attached to the scenes that I was witnessing”.

Others described pronounced *ego-loss*, a loss of the boundaries of the ordinary self and a sense of merging with the surrounding environment:

“The concrete borders of my sense of self were obliterated. I became part of everything. Who I was was centered in my heart and was projected into the universe”.

Several accounts involved a felt sense of *alignment across aspects of the self*, in which previously fragmented or disowned parts felt reconciled:

“I feel that my conscious and subconscious are now aligned”.

Experiences of *mystical connection* were also prominent. Participants described moments of contact with a divine presence or universal consciousness, often accompanied by awe:

“I met God and had a conversation with him. I asked, ‘Who are you?’ God: ‘I am you’”.

These states were frequently tied to a renewed sense of *meaning*, purpose, or existential clarity:

“It was revealed that the purpose was to experience pure love and what we should share with our fellow life on earth, in the heaven that was created for us”.

### Emotional resolution and compassionate reconnection

Emotional breakthroughs were widespread. Many described a *rediscovery of love*, characterized by sudden, overwhelming feelings of connection to family, friends, and community:

“All the pain came out. Love for family and friends rushed in. It was overwhelming and lasted several minutes”.

Participants also reported profound *compassion* and *forgiveness*, both toward themselves and others. One participant wrote:

“I was taken to my audit in which an angel ran me through my entire life and all the events which burdened my soul…I realized the events were being shown not to make me feel shame or guilt, but to forgive myself as God had forgiven me”.

### Embodied brain healing and after effects

Many participants described somatic sensations they interpreted as *brain healing*, often involving vivid perceptions of neural activity or synaptic reorganization:

“What was happening in my brain was as if all my neurons and synapses were firing and recharging and healing”.

Beyond the acute experience, participants reported a variety of enduring *post-treatment effects*, including reduced cravings, improved attention, improved mood, and changes in physical or behavioral habits:

“What HAS changed is that my coffee habit of several liters per day for the past 20+ years is gone … I had a glass of wine, and was repulsed by the way it made my head feel”.

## Discussion

Employing an inductive grounded‑theory framework, we captured the lived phenomenology of participants as they progressed through this rapid course of psychological recovery. In doing so, a unifying theme emerged: subjectively, ibogaine resembled an accelerated, self-guided psychotherapeutic process, in which traumatic memories resurfaced, were re-examined from novel vantage points, and were re-integrated with intensified feelings of love, forgiveness, and purpose. We use the term “auto-psychotherapy” descriptively to characterize how participants themselves portrayed their experiences: namely, as a condensed, self-directed process of emotional insight and resolution. With ibogaine, accelerated auto-psychotherapy mostly occurred within a single session. We recognize that similar terminology appeared in early psychedelic literature, sometimes implying an autonomous or self-curative process. Our intent here is more limited: to capture the subjective phenomenology of participants’ perceived self-guided therapeutic work, rather than to assert equivalence with structured psychotherapy or to make clinical efficacy claims. In this sense, the phrase serves as a heuristic label for a lived experiential process rather than a prescriptive therapeutic construct. Nevertheless, these results suggest that the subjective qualities of ibogaine treatment may be integral to the impressive clinical improvements previously reported in the same cohort. Below, we interpret the four domains through a neurophenomenological lens, mapping subjective themes to psychotherapeutic mechanisms, contextualizing them within the psychedelic literature, and linking them to candidate neural processes.

Consistent with earlier accounts of ibogaine’s capacity to evoke a symbolic, emotionally rich, and autobiographical state^10,11^, participants in our study repeatedly described experiences likened to waking dreams (i.e., ‘oneiric’). Such experiences plausibly explain the frequent reports of dialogue with beings perceived as external to the self, as this is a core feature of natural dreaming. Preclinical work in rodents has shown that ibogaine elicits a distinct waking neurophysiological state, characterized by heightened gamma power alongside reduced network coherence and complexity, an oscillatory signature reminiscent of REM sleep^23^, the stage of sleep most strongly associated with dreaming. Interestingly, recent case reports have shown that dreams induced under anesthesia may confer comparable therapeutic benefits for trauma processing^24^ with striking qualitative parallels to the accounts in our study. A growing body of literature further demonstrates that dreams contribute to the strengthening, transformation, or attenuation of emotionally salient memories^25^. This convergence raises the possibility that ibogaine’s phenomenology and potential therapeutic benefits may emerge, at least in part, from neural mechanisms overlapping with the dream state.

The converging phenomenological and neurobiological parallels between dreaming and the psychedelic state have attracted growing attention in recent work, particularly in relation to the REBUS (Relaxed Beliefs Under Psychedelics) account of weakened high‑level priors^26–31^. REBUS proposes that psychedelics make the brain’s high‑level predictions less rigid, permitting suppressed or emotionally charged material to surface^26,28,29,32^. Dreams display a comparable pattern, with spontaneous, unconstrained activity less governed by rigid predictive models^27,28^.

Understanding ibogaine’s pharmacology reinforces this interpretation. Unlike the classical psychedelics, which primarily exert effects via 5-HT2A receptor agonism, ibogaine shows minimal action at this site^9^ and instead engages alternative mechanisms, including kappa-opioid receptor agonism^33^. Both REM sleep and kappa agonists, such as salvinorin A, have been shown to reduce top-down priors by disrupting default-mode network (DMN) connectivity^27,34^. In line with REM physiology, this diminished top-down cortical control may facilitate the resurfacing and integration of suppressed autobiographical material, echoing Freud’s injunction to bring unconscious content into awareness^28,35^ and to reintegrate repressed elements of the psyche^36^. Although the concept of repression remains debated, several participants described the resurfacing and emotional resolution of previously avoided or inaccessible traumatic material. We use the term “repression” here in a phenomenological rather than literal sense—to denote the subjective experience of integrating painful content that had been defended against awareness (one of PTSD’s cognitive alteration symptoms)^37^. The ongoing “false memory” debate highlights the complexity of this issue, suggesting that both genuine recovered memories and false recollections can occur, as reviewed in Farrants et al. (1998)^38^. Within the context of psychedelic-assisted psychotherapy, such processes may reflect a relaxation of defensive priors and the reintegration of previously excluded content, consistent with both psychodynamic and predictive-processing frameworks of therapeutic change. In addition to repression, several participants in our study also spoke of reconciling disparate facets of their psyche, evocative of Freud’s aspiration to balance id, ego, and superego^39^—a cornerstone of psychodynamic thought^40^. Concerning our theme of insight, participants in our study frequently reported clear, objective recognition of previously ego-defended behaviors and their consequences. This aligns with emerging models that link insight to the relaxation of high-level ego defenses^32,41,42^.

Beyond the Freudian framework of ego mediation and defensive processes, the ibogaine experience can also be considered through a Jungian lens. Jung’s model distinguishes between the subjective consciousness and the collective unconscious, emphasizing the transcendent function—the integrative process by which previously unconscious material becomes available to awareness, promoting psychological wholeness or individuation^43^. Many participants’ accounts of symbolic death, renewal, and encounters with archetypal figures (e.g., divine or ancestral presences) echo these Jungian themes^44^. The numinous and universally patterned qualities of these experiences align with Jung’s notion of the archetypal, providing a vocabulary for understanding mystical and symbolic content reported by participants in this study. From this perspective, ibogaine may facilitate a rapid convergence between conscious and unconscious aspects of the psyche (whether individual or collective), paralleling the goal of Jungian analysis to restore dialogue between these domains.

Building upon this idea, participants also described profound alterations in their sense of self, including experiences of ego dissolution, out-of-body sensations, and feeling as though they were an “un-attached observer.” This description of experiencing themselves from a neutral, third-person stance is reminiscent of mindfulness practices that cultivate non-judgmental, present-moment awareness^45^ and of Acceptance and Commitment Therapy (ACT), which trains a “self-as-context” perspective that decouples thoughts from identity^46^. Comparable phenomena are also seen in clinical hypnosis for trauma therapy, which often looks to induce dissociation, a “suspension of self”, and altered agency^47–50^, thereby facilitating cognitive restructuring of traumatic memories^51^. Research shows suppression of DMN activity during hypnosis^51^, which may help to explain the dissociative capacity to ‘try out being different’ in hypnosis by suppressing the connection between conscious mental activity and customary awareness of self. Notably, similar ego-distancing and fear attenuation have been observed during MDMA-assisted psychotherapy, where reduced amygdala reactivity is linked to diminished threat perception and enhanced emotional processing^52^. Such detachment may have allowed the participants in our study to revisit traumatic material without the usual negative self-critical emotional valence^53^, a central barrier to standard trauma therapy. Ibogaine’s NMDA-receptor antagonism^54^ may add a ketamine-like dissociative component^55^, corresponding with the out-of-body sensations reported here.

Throughout the psychedelic literature, alterations in the sense of self and ego-loss have been associated with a weakening of high-level cognitive frameworks, such as the DMN, that maintain the sense of self^56^. Crucially, altered self-perception is highly linked to mystical or transcendent experiences^57–59^. In our study, some participants recounted ego death, encounters with a greater presence, and an overwhelming sense of interconnectedness. These experiences parallel those elicited by classic psychedelics like psilocybin^60^, underscoring ibogaine’s capacity to provoke similarly transformative states. Mystical phenomena have long been recognised as powerful therapeutic catalysts, from William James’s *Varieties of Religious Experience*^61,62^ to Maslow’s peak experiences^63^. Additionally, a recent systematic review provided evidence that mystical experiences may be linked to the clinical benefits of psychedelic substances^8^. Our quantitative data from this same cohort mirror this pattern: higher mystical‑experience scores correlated with greater reductions in PTSD symptoms^7^. Moreover, participants in our study frequently reported deriving profound meaning from their experiences, describing a symbolic “death” to their old selves and the emergence of renewed purpose. This meaning‑making resonates with existential psychotherapies, such as Viktor Frankl’s logotherapy, which views purpose as a central driver of psychological healing: “One can endure almost any ‘what’ if one has a ‘why.’”^64^ Such existential therapies have also shown robust clinical benefits^65^.

In parallel with these existential and mystical transformations, participants also described powerful emotional reconnections, marked by profound experiences of empathy, forgiveness, and love. These reports also parallel the emotional tone seen in MDMA^66^. From a psychotherapeutic perspective, their frequent sense of being supported or accepted by loved ones recalls parallel ideas from humanistic psychotherapy, wherein Carl Rogers championed unconditional positive regard and empathy as cornerstones of psychological healing^67^. Indeed, large psychotherapy datasets identify therapist empathy as a leading predictor of clinical outcome^68^. Remarkably, ibogaine appears to generate an analogous empathic climate intrinsically, perhaps substituting for an external therapeutic alliance. *(*See Supplementary Information 3 for further discussion of the psychotherapeutic background).

Participants’ descriptions of enhanced empathy, self-understanding, and insight into the perspectives of loved ones may reflect transient increases in mentalization—the capacity to perceive and interpret the mental states of oneself and others. In contemporary psychotherapy, this capacity is central to Mentalization-Based Therapy^69^, where reflective awareness and perspective-taking support emotional regulation and interpersonal skills. The altered states of consciousness elicited by ibogaine may transiently soften rigid self-boundaries, fostering a sense of interconnectedness and empathy that enables individuals to appreciate others’ inner worlds as distinct from their own. Simultaneously, the emotionally “softer,” more flexible processing of previously defended or psychosomatic reactions may allow traumatic material to be revisited with curiosity and compassion rather than fear or avoidance. As with hypnosis, the capacity to experience a situation or memory from a different point of view can have the effect of enhancing the ability to imagine others’ differing legitimate perspectives on oneself. In this way, ibogaine may momentarily recreate conditions similar to those cultivated through long-term mentalization-based or compassion-focused therapies, promoting both self-reflection and prosocial reconnection.

The reappraisal observed here resembles the restructuring of maladaptive beliefs targeted in cognitive behavioral therapy (CBT). In standard CBT, core beliefs shape automatic thoughts, thereby influencing emotion and behavior^70^. Shifting such beliefs typically demands extended work, as therapists guide clients to recognize evidence contradicting entrenched assumptions. In our ibogaine cohort, by contrast, participants experienced potent episodes of pro‑social love or feelings of being loved that directly undermined negative core beliefs. This immediate experiential evidence may have precipitated rapid changes in automatic thoughts and actions, effectively compressing the cognitive restructuring central to CBT. Reported experiences also echoed exposure‑based processes, wherein traumatic memories are revisited under conditions of agency and felt safety, promoting extinction learning^71^. Given meta‑analytic support for CBT as the most effective treatment for PTSD^72^, these parallels have clear clinical salience.

While the present analysis is qualitative, a brief reference to neurobiological models may help contextualize the phenomenology and generate hypotheses for future empirical study. At the neurochemical level, ibogaine’s principal metabolite, noribogaine, inhibits the serotonin transporter (SERT)^73^, which may elevate serotonin availability and contribute to the anxiolytic or mood-stabilising effects often reported following treatment^74,75^. While in-session neuroimaging data on ibogaine are not yet available, the phenomenology described here—including diminished fear responses and heightened feelings of social connectedness—may be consistent with transient modulation of limbic circuits such as the amygdala, similar to what has been seen with MDMA^76^. Ibogaine’s engagement with serotonin receptors, such as 5‑HT2C and 5‑HT1A^77^, may also stimulate oxytocin release^78^, a hormone highly associated with facilitating social bonding^79^.

Beyond psychological restructuring, participants frequently described vivid sensations of “brain healing” or neural “reset,” often accompanied by imagery of electrical activity, cleansing, or reorganization within the head. These experiences should be understood primarily as embodied metaphors of psychological and physiological restoration. Nevertheless, interestingly, this same cohort was later shown to exhibit significant increases in cognitive processing speed and executive function, as well as decreases in standardized measures of disability^2^. While these convergences do not imply that participants were literally feeling neurobiological repair, they invite an integrative interpretation—one in which the subjective perception of healing may mirror, symbolically or indirectly, processes of neural reorganization occurring at other levels of analysis. Future research combining first-person phenomenology with longitudinal imaging could help clarify how such experiential metaphors relate to measurable changes in brain function.

Interestingly, several of the themes identified here—such as autobiographical reappraisal, emotional catharsis, and the sense of renewed meaning—resonate with early descriptions of classic psychedelic experiences from the mid-20th century^80^. Rather than detracting from ibogaine’s distinctiveness, this overlap may point to shared psychological mechanisms that emerge when rigid cognitive and emotional patterns are relaxed, regardless of the compound’s primary receptor targets. In this sense, ibogaine appears to occupy a unique pharmacological position: unlike the purely serotonergic psychedelics, it also engages NMDA and kappa-opioid systems and produces empathogenic and dream-like qualities reminiscent of both MDMA and dissociative agents. Its ability to evoke processes characteristic of several psychotherapeutic drug classes may help explain why participants describe it as simultaneously introspective, emotionally connective, and reparative. Taken together, our findings position ibogaine as a unique oneirogenic agent that appears to compress established psychotherapeutic processes and neurorestoration into a single, self-guided session, offering a promising blueprint for accelerated trauma therapies.

Several factors temper our conclusions. The parent trial was open-label, uncontrolled, and observational, focused on a novel indication for ibogaine (i.e., TBI). Our qualitative analyses were retrospective, and participants wrote their narratives up to two weeks post-dosing, introducing recall bias. Further risk for bias may come from the qualitative interpretation by the study team, and while efforts were made to minimize it, risk for bias still exists. All responses were self-written rather than interview-based, limiting our ability to probe unclear statements or non-verbal cues. Finally, the cohort consisted exclusively of male Special Operations veterans, which constrains generalisability.

## Supplementary information

Supplementary materials

## Data Availability

The data analyzed in this study consist of confidential participant narratives and are not publicly available owing to privacy restrictions. De-identified thematic data may be shared by request with appropriate ethical approval.
