# Supplementary material for: Accelerated recovery using magnesium ibogaine: characterizing the subjective experience of its rapid healing from neuropsychiatric disorders
Source: Npj Ment Health Res. 2026 Jan 31;5:8. doi: 10.1038/s44184-026-00185-7 (PMC12860795; doi:10.1038/s44184-026-00185-7)
Supplement: Supplementary file 1 — Supplementary materials [file 44184_2026_185_MOESM1_ESM.pdf]

Supplementary Materials

Supplement 1: Questions that the veterans responded to.

Question 1: Mystical Experiences

In your own words, please describe your experience with Ibogaine. We understand that many of the experiences you had may be hard to describe or indescribable. However, we would like you to try your best to put your experience into your own words. Start from the beginning of your experience and retell what occurred. Please be sure to include any notable flashbacks, visual or auditory hallucinations, and confrontations with past unresolved conflicts. If you confronted any past unresolved conflicts, what occurred when you encountered them? If the issue was resolved, how did this occur? Also, please be sure to mention any changes in your sense of self and time throughout your experience.

Question 2: Sense of Time

Please describe below any changes in your sense of time. Did time feel altered in any way? If so, was it sped up or slowed down? Did you ever feel as though you were outside of time? If so, please describe how this felt. Use as much detail as possible.

Question 3: Sense of Self

Please describe below any changes in your sense of self. Did you feel as though your sense of self had changed in any way throughout your experience? To what extent did you feel like your sense of self was located behind your eyes (i.e. in your head) vs. centerless/at no particular location? To what extent did you feel like there were concrete boundaries between yourself and the outside world?

Supplement 2: Additional participant quotations for each theme: This is not exhaustive.

Trauma Processing and Dialogic Insight

Re-encounter/Re-appraisal of Trauma:

> "During my journey on Ibogaine, I was able to uncover something that I had buried so deep that decades of therapy with the best therapists in the world would have never uncovered…. My mind blacked it all out except for a couple of fleeting memories in order to survive. I had carried this burden around with me for over 30 years, not knowing what was wrong with me, and it was the cause of my drinking to excess and indulging in food in order to cope. After Ibogaine, all the past trauma associated with that event is gone. The weight of the world on my shoulders is gone. The feeling that my heart is being crushed in a vise is gone. I am totally at peace in this world and am finally able to get a good night's rest and awaken happy and excited to start the day. Ibogaine saved my life!!!"

> "My mind made me work through a recurring horrific scene of a child being killed until I was able to rescue the child."

> "He was also my best man at my wedding. He has been depressed and sometimes suicidal since we were young. He drank, he drank a lot. I tried to get him to come live with me [REDACTED]. He was too far gone. I spent a lot of time on the phone with him. I thought I could save him. I could not. He committed suicide …. It was always expected, but it still hit me hard. I could not even do the eulogy; I was in too much pain to talk by then. In the vision, he was smiling and happy. I felt better."

Therapeutic Dialogue:

> "The voice that spoke to me in the darkness spoke out again, asking me if I understood the lessons I was being provided."

> "Sometimes I would ask it [ibogaine] questions, and sometimes it would show me images to help me see the answer."

> "My subconscious and I were making jokes to each other all night. It was really enjoyable, he is hilarious."

> "In my head, I remember thinking that the medicine was discovering and learning me and preparing itself for the journey."

> "The medicine "told me" It needed the water out [to purge: emesis] before we could begin."

Insight:

> "I saw myself in a car driving next to a woman in a car. She cut me off, and so I cut her off out of anger. I watched as she cut off two more people…until my one act of anger infected the entire planet as it caused everyone in the world to perform acts of anger towards each other. I watched as the globe turned black from all this evil. I thought about

> the power of one act of anger for a while and eventually wondered: what if instead of an act of anger I performed an act of kindness? Something unexpected and surprising. Could it lead to a domino effect of everyone in the world performing acts of kindness, and could that one act be powerful enough to fill the world with joy?"

> "Eventually, I was shown my family being scared around me, which was hard to watch but necessary for me to realize the negative energy I was filling my home with."

> "I asked how I could be a better husband to my wife. I had a long, deep conversation with my higher consciousness on this. We went around and around, and eventually, I had the realization that I need to love her without guilt."

> "I asked how I could be a better father to my sons. I watched myself interact with them and become frustrated and snap at them. I could see how counterproductive and mean the way I handled the situation was. I could see how the only thing they were learning was that I was an angry dick. I reflected on the interaction I had watched with guilt and shame. My higher conscience asked me to do the situation again, this time with me in it. I handled it this time by taking a breath, remaining calm, and discussing what I was seeing and why I found it upsetting. I explained to the boys, with love and concern, why what they were doing was wrong. I made sure they understood without losing my temper or raising my voice. After, my higher conscience asked me how I felt about that interaction. It felt great and we high-fived."

Altered Self and Mystical Connection

Unattached Observer/Out of Body:

> "It felt more like my ego was suspended, and I was completely a witness and not attached to the scenes that I was witnessing. Even the horrific scenes of seeing [REDACTED; teammate 1 name] suicide didn't seem to faze me. I was just an unattached observer witness to the scene."

> "I felt like it was me separate from my thoughts, sounds, or what I was seeing or experiencing. It was like I was this little innocent soul that was observing all of these things going on around me…It's hard to explain, but there was a definite separation within myself."

> "After taking some time to explore my new self and reflect on the journey my treatment took me on, I found that I had been exposed to any and all traumas that I held guilt or shame or resentment or pain for, and was allowed to look at myself and others from God's perspective."

Ego-loss:

Ego loss refers to instances where participants felt that their normal sense of 'self' disintegrated and they were unshackled from their physical body.

"My sense of self was not just behind my eyes during this journey. It did seem centerless and had no particular location at all. I felt like there were no concrete barriers whatsoever between myself in the outside world. I really felt that anything was possible."

"The concrete borders of my sense of self were obliterated. I became part of everything. Who I was was centered in my heart and was projected into the universe."

Aligning Selves:

"I feel that my conscious and subconscious are now aligned."

"it became clear that my spiritual self included my nervous system and that was a separate entity than my physical body, which has its own 'mind' that felt like it was in my gut. I was shown how the goal in life is for these two parts of our existence to merge into one higher self once you learn to truly love yourself and treat your soul and body as best that you can."

Mystical Connection:

"I met God and had a conversation with him. I asked, "Who are you?" God: "I am you." I responded, "Then who am I?" God: "You are me."

"My connection with the divine is more real and tangible than I could have imagined. I know the limits of my imagination, and this experience was far beyond anything that my own mind could have created. My consciousness had expanded so much that I could feel the overwhelming energy that binds every atom together in our universe, and I knew that it was all intelligently designed that way."

"I was shown [the] creation of the universe, multiple worlds, and how all life was created and is connected."

"The universe appeared to me under my eyeshades; I could see the stars and heavens as if I were outside looking at the sky."

"I had an overwhelming feeling as if a higher power or someone of reverence very authoritatively said to the effect of 'You've received what you need, leave this in the past and move forward."

Meaning Making:

"It was revealed that the purpose was to experience pure love and what we should share with our fellow life on earth, in the heaven that was created for us. To embrace love again and what my true purpose was in life."

"Then it shifted to some scenes of me doing talk therapy with some young team guys. It shows several scenes of me as the therapist talking to these guys and having a very positive impact. I was kind of blown away by this at first. It showed how I deeply affected their lives in my life as well. This went on for a while it was clear to me that this has the potential to impact so many lives. I feel a deep sense of calm and gratitude at

these images. It brought back some scenes of me when I first started going to talk therapy and how frustrated I was at being able to find somebody that I could connect with. It showed not only the impact that I had on the young team guys but the impact it had on their children and their families, and their community at large. It showed an even older version of myself really enjoying this work and getting really a lot of satisfaction out of it."

Emotional Resolution and Compassionate Reconnection

Rediscovery of Love:

"All the pain came out. Love for family and friends rushed in. It was overwhelming and lasted several minutes."

"I found that the immense and crippling self-hatred and guilt and shame that I carried with me for so long had also morphed into love and understanding for myself."

"I fell asleep, but was not asleep. I felt warm, my loving hands and whispers of prayer and love from people supporting me and angels and past loved ones, including my grandmother, and that I was safe and secure. Then the magic really happened."

"All the love. I realized how much feeling I had lost from the war in a flash that was overwhelming. Again, it was so beautiful that my head cocked back, mouth open, howling because the love I felt was so intense."

Compassion/Forgiveness:

"He climbed up and sat on the rockpile. I said "I'm sorry" to him. He said, "I was trying to kill you and your men, you are a soldier, you were protecting your men". Then he disappeared. I felt forgiven. I never expected that, nor did I think that he would appear. I felt very good. No fear."

"I was surrounded by my teammates who had died over the years. They reached down and picked me up, and I spent time hugging them, crying, apologizing, and felt their love and grace. Some gave me messages to deliver to family members."

Embodied Brain Healing and After Effects

Felt Brain Healing:

"I felt what was similar to electric pulses going through my entire body. It felt like they were synapses that were purging my nervous system, relaxing my muscles, and realigning my skeletal system."

> “I could feel my brain healing. I could feel that powerful medicine making my brain whole again. It was not unpleasant. It felt great. Soothing. The medicine was nourishing my brain, and I was feeling the awesome effects as it happened. I could visualize it happening. Smoke or steam was leaving the top of my head.”

> “I felt what was similar to electric pulses going through my entire body … realigning my skeletal system.”

Post-Treatment After-Effects:

> “I was also relieved to find that my splitting migraine headache that had been my constant companion since [REDACTED: date; duration of chronic migraines] was completely gone, as was most of the other pains and aches I had grown accustomed to.”

> “I haven't slept like this in forever, anxiety is lessened a lot, anger is not as prevalent.”

> “Before ibogaine, I would've had a hard time accessing thoughts and information during a conversation. I would also forget things. Like, what did I just walk here for, etc. Now my thoughts flow right to my verbal expression. It's awesome to have my thoughts flowing freely and be able to express myself really well.”

Supplement 3: Additional context for the discussion around accelerated auto-psychotherapy and Constructivist Grounded Theory.

Cognitive-Behavioral Therapy (CBT)

Cognitive-behavioral therapy (CBT) is a widely used psychological treatment based on several fundamental principles focusing on the interconnections between thoughts, feelings, and behaviors. CBT posits that maladaptive cognitive patterns often fuel negative emotions and behaviors and that by challenging these maladaptive cognitive patterns, patients can improve their emotional and psychological lives.[1] CBT is one of the most effective therapies for treating substance use disorders and a broad range of mental health disorders.

Another central tenet of CBT is exposure therapy, in which patients are repeatedly exposed to fear-inducing stimuli in a safe setting. Over time, patients develop new and less threatening associations with the formerly feared stimuli, which override the old, maladaptive learning. [2]

Psychodynamic Psychotherapy

Psychodynamic therapy, originating from the work of Sigmund Freud, is a therapy that is predicated on the exploration of unconscious wishes, desires, and thought processes. [3] The theory posits that unconscious desires, often originating from traumatic or stressful childhood events, subconsciously influence patients' behavior and mental health. It emphasizes the therapeutic relationship as a means to unearth and resolve these subconscious psychological desires, generally by revisiting these seminal events from childhood.[4] By revisiting past events, the hope is to bring them into awareness and reintegrate them with the conscious psyche. The therapy also includes many now-discredited ideas about libido and psychic energy; however, the core idea remains the exploration of the unconscious mind and its impact on psychological health. [5]

Existential/Humanistic Psychotherapy

Existential psychotherapy, rooted in existential philosophy, is a therapy that focuses on the individual's confrontation with central aspects of human existence, such as death, freedom, isolation, and the search for meaning. Pioneers of this theory, such as Viktor Frankl and Irvin Yalom, argued that psychological dysfunction often arises from a failure to truly engage these key aspects of existence, which can morph into feelings of alienation, isolation, and meaninglessness. [6]

Humanistic psychotherapy emerged in the 1950s as a response to the overly deterministic and negatively biased views of psychoanalytic theory. Led by figures such as Carl Rogers and Abraham Maslow, this approach focuses on seeing individuals as wholes rather than parts and bringing out the positive aspects of each person's true nature.[7] Humanistic psychotherapy is known for its optimistic view of human nature and emphasizes the importance of a supportive and empathetic relationship with others.

Clinical Hypnosis

Clinical hypnosis (also termed "hypnotherapy") is a psychological technique utilizing

verbal suggestions as a means for facilitating clinical change to address medical or psychological concerns. [8] In the context of hypnosis, suggestions are made to elicit alterations in perceptions, cognitions, emotions, and behaviors. Hypnosis has long been associated with dissociation [9], alterations of agency [10], and other modulations of conscious experiences. [11] Recent reviews highlighted the qualitative similarities between hypnosis and psychedelics and suggested a psychotherapeutic potential in combining the two approaches. [12,13] Studies of changes of brain activity during hypnosis indicate reductions in activity in the salience network, especially the dorsal anterior cingulate cortex, accounting for an ability to reduce psychophysiological arousal, and also inverse functional connectivity between the executive control network and the posterior cingulate cortex, leading to what has been termed a ‘suspension of self. [14] This facilitates dissociation from and affective and cognitive restructuring of responses to traumatic memories.[15]

Ibogaine and Memory Reconsolidation and Psychotherapy

Research from Lane et al. (2014)[16] has shown that therapeutic change in modalities like behavioral, cognitive-behavioral, emotion-focused, and psychodynamic psychotherapy results from updating prior emotional memories through reconsolidation and incorporating new emotional experiences. The authors propose that effective therapy involves reactivating old memories, integrating new emotional experiences during reconsolidation, and reinforcing these changes through new behaviors and experiences in different contexts. This integrated model, proposed to explain memory reconsolidation in traditional psychotherapeutic practices, clearly relates to the thematic ideas seen in our study.

References

1. Beck, J. S. *Cognitive Behavior Therapy, Second Edition: Basics and Beyond*. (Guilford Press, 2011).
2. Foa, E. B. & Kozak, M. J. Emotional processing of fear: Exposure to corrective information. *Psychol. Bull.* **99**, 20–35 (1986).
3. Jones, J. B. Freud, Sigmund. in *The Encyclopedia of Literary and Cultural Theory* (John Wiley & Sons, Ltd, 2010). doi:10.1002/9781444337839.wbelctv1f005.
4. Chessick, R. D. *The Future of Psychoanalysis*. (State University of New York Press, 2012).
5. Shedler, J. The efficacy of psychodynamic psychotherapy. *Am. Psychol.* **65**, 98–109 (2010).
6. Yalom, I. D. *Existential Psychotherapy*. (Hachette UK, 2020).

7. Schneider, K., Pierson, J. & Bugental, J. *The Handbook of Humanistic Psychology: Theory, Research, and Practice*. (SAGE Publications, Inc., 2455 Teller Road, Thousand Oaks California 91320 United States, 2015). doi:10.4135/9781483387864.
8. Elkins, G. R., Barabasz, A. F., Council, J. R. & Spiegel, D. Advancing research and practice: The revised APA division 30 definition of hypnosis. *Am. J. Clin. Hypn.* **57**, 378–385 (2015).
9. Spiegel, D. Hypnosis, dissociation and trauma. *Int. Handb. Clin. Hypn.* 143–158 (2001) doi:10.1002/0470846402.ch10.
10. Polito, V., Barnier, A. J. & Woody, E. Z. Developing the Sense of Agency Rating Scale (SOARS): An empirical measure of agency disruption in hypnosis. *Conscious. Cogn.* **22**, 684–696 (2013).
11. Terhune, D. B., Cleeremans, A., Raz, A. & Lynn, S. J. Hypnosis and top-down regulation of consciousness. *Neurosci. Biobehav. Rev.* **81**, 59–74 (2017).
12. Lemercier, C. E. & Terhune, D. B. Psychedelics and hypnosis: Commonalities and therapeutic implications. *J. Psychopharmacol. (Oxf.)* **32**, 732–740 (2018).
13. Timmermann, C. *et al.* A neurophenomenological approach to non-ordinary states of consciousness: Hypnosis, meditation, and psychedelics. *Trends Cogn. Sci.* **27**, 139–159 (2023).
14. Jiang, H., White, M. P., Greicius, M. D., Waelde, L. C. & Spiegel, D. Brain activity and functional connectivity associated with hypnosis. *Cereb. Cortex N. Y. N 1991* **27**, 4083–4093 (2017).
15. Spiegel, D. Tranceformation: Digital dissemination of hypnosis. *Neuron* **112**, 340–341 (2024).
16. Lane, R. D., Ryan, L., Nadel, L. & Greenberg, L. Memory reconsolidation, emotional arousal, and the process of change in psychotherapy: New insights from brain science. *Behav. Brain Sci.* **38**, (2014).
